# Effective Treatment of Acinetobacter baumannii Ventriculitis With Interventricular Colistin: A Case Report

**DOI:** 10.7759/cureus.62169

**Published:** 2024-06-11

**Authors:** Ithamar Cheyne, Kamelia Hassan, Tjard Dunkel, Marcin Sota, Łukasz Wróblewski, Małgorzata Mikaszewska- Sokolewicz

**Affiliations:** 1 Anesthesiology and Intensive Care Scientific Circle English Division (ANKONA ED), Medical University of Warsaw, Warsaw, POL; 2 2nd Department of Anesthesiology and Intensive Care, Medical University of Warsaw, Warsaw, POL; 3 Department of Anesthesiology and Intensive Care, Children's Memorial Health Institute, Warsaw, POL

**Keywords:** drug administration routes, treatment outcome, case report, intraventricular, colistin, ventriculitis, acinetobacter baumannii

## Abstract

Cerebrospinal fluid shunts are the primary treatment for hydrocephalus. However, prolonged external ventricular drain (EVD) use can lead to central nervous system (CNS) infections such as ventriculitis. In the ICU setting, nosocomial infections with gram-negative, multi-drug resistant (MDR) organisms such as *Acinetobacter baumannii* (AB) prevail, leading to poor outcomes. AB infections are notably challenging due to their genetic drug resistance. Colistin has been reintroduced for use against gram-negative MDR pathogens but has limitations in CNS penetration when administered intravenously. Therefore, intraventricular (IVT) or intrathecal administration of colistin is recommended to enhance its therapeutic reach within the CNS.

We present a case of a 22-year-old male admitted after an electric scooter accident with head trauma and hydrocephalus. A ventriculoperitoneal (VP) shunt was inserted, complicated by a nosocomial neuroinfection. Empiric IV therapy with meropenem and vancomycin was initiated. The VP shunt culture identified AB susceptible only to colistin. Intravenous (IV) colistin was added to meropenem with no significant improvement. The addition of IVT colistin significantly improved the patient's neurological condition and reduced inflammatory markers. The patient experienced one myoclonic seizure during IVT colistin treatment, managed with antiepileptics. After multiple unrelated nosocomial complications, the patient was discharged in good condition to rehabilitation.

This case suggests that IVT colistin, combined with IV administration, may be preferable over IV colistin alone. Medical staff should be informed about the correct prevention and care of EVD-associated infections.

## Introduction

Cerebrospinal fluid (CSF) shunts serve as the primary treatment for hydrocephalus, offering both effectiveness and relative safety [1]. However, using external ventricular devices (EVD) elevates the risk of central nervous system (CNS) infections, with incidence rates reaching 18%, varying depending on patient comorbidities and associated risk factors [2,3]. The most common pathogens responsible for these CNS infections are typically gram-positive bacteria originating from skin flora, but in the intensive care unit (ICU) setting, gram-negative, multi-drug resistant (MDR) organisms prevail [4].

Of growing concern is the emergence of *Acinetobacter baumannii *(AB) as a significant nosocomial infectious threat, particularly affecting patients undergoing invasive procedures such as EVD placement [5,6]. Notably, infections caused by AB pose a formidable challenge due to their remarkable genetic drug resistance profile, compounded by rising resistance to commonly available antibiotics [7].

The reintroduction of colistin into clinical practice has provided a valuable treatment option for MDR gram-negative infections [8]. However, its efficacy is tempered by pharmacokinetic limitations, particularly in its ability to penetrate to the CNS when administered intravenously. Consequently, intraventricular (IVT) or intrathecal (ITH) administration of colistin is warranted to enhance its therapeutic reach within the CNS.

In this case, we suggest opting for IVT colistin in combination with IV colistin to treat susceptible AB ventriculitis.

## Case presentation

**Figure 1:**
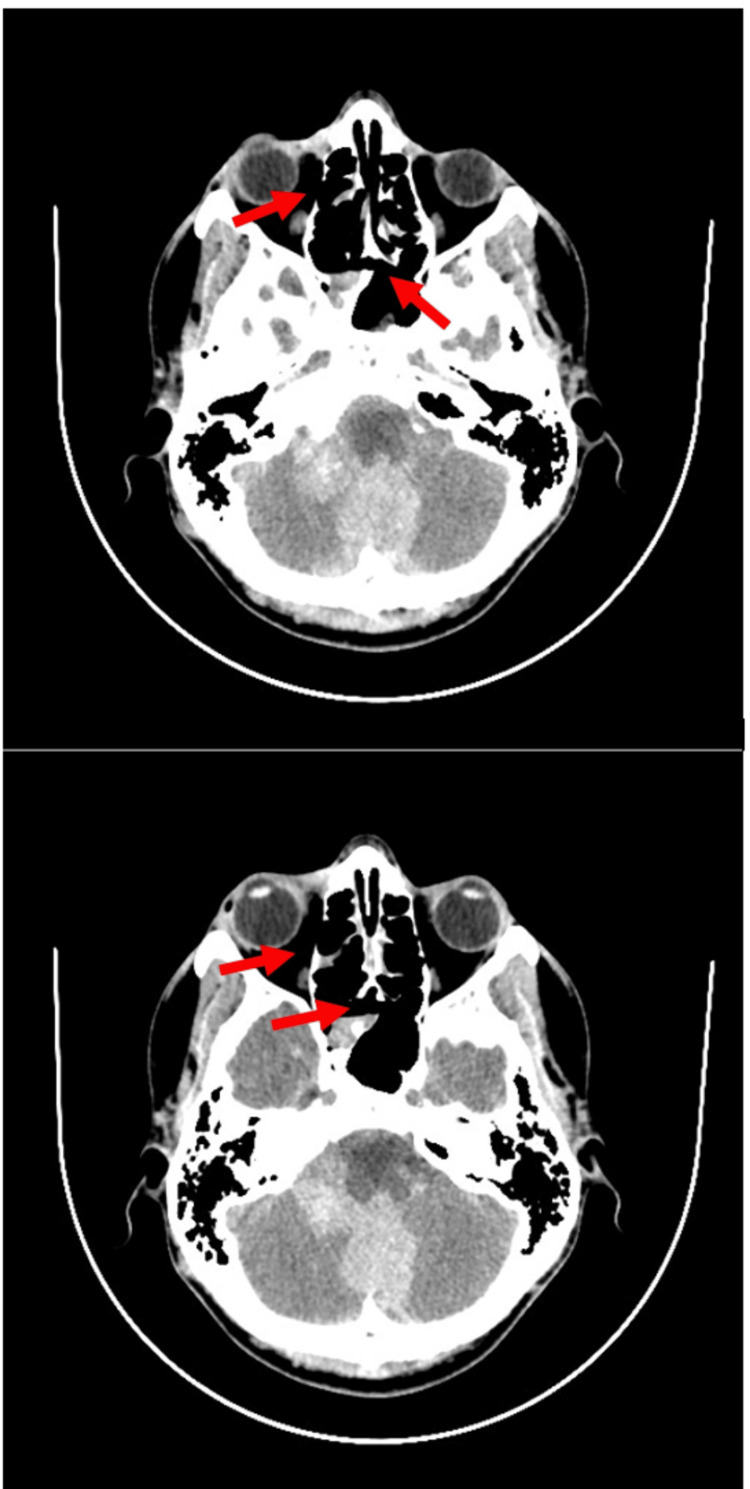
CT scan from the day of admission showing multiple facial fractures (top) and subdural hematoma (bottom) due to scooter accident

A 22-year-old head trauma victim was admitted from a lower reference hospital. The patient rode an electric scooter while under the influence of alcohol, fell off, and suffered a cerebral contusion and multiple facial fractures. Apart from the latest injury, the patient was generally healthy and had no relevant medical history. A CT scan showed subdural hematoma as well as multiple facial fractures; multi-organ trauma was excluded (Figure 1). The patient was hospitalized in the ICU, where he was treated for his injuries. On the fourth day of hospitalization, he experienced a CNS infection. The causative pathogen was not identified, and the patient was treated with multiple antibiotic regimens until the infection was clinically resolved on day 34 of the hospitalization.

On day 35 of hospitalization, the patient successfully underwent a lumboperitoneal shunt insertion, during which a CSF sample was collected. The results showed no signs of inflammation or abnormalities. Post-operation, the patient remained hemodynamically stable and conscious, albeit with mildly delayed responses to questioning and a mild headache.

**Figure 2:**
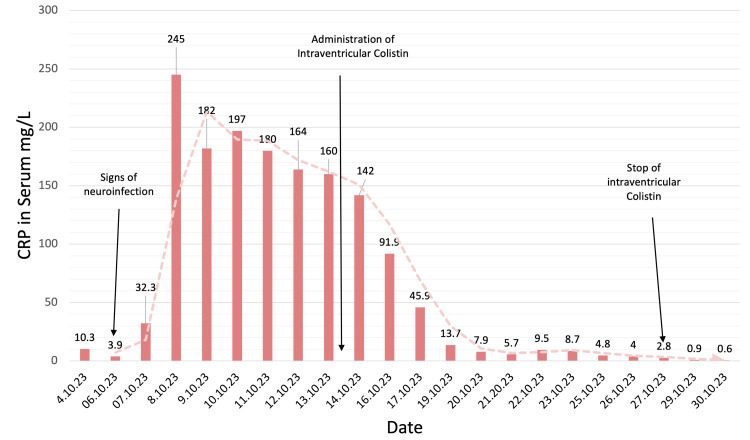
CRP levels in serum. High CRP levels due to a neuroinfection were only reduced after the dual therapy of IV and IVT colistin treatment CRP, C-reactive protein; IV, intravenous; IVT, intraventricular

On the following day, a neuroinfection was suspected due to diplopia, headaches, deterioration in activity level, and severe neck stiffness. Increased levels of C-reactive protein (CRP), white blood cell (WBC) count, and procalcitonin (PCT) levels (Figure 2, Table 1) were observed. On the same day, blood and urine cultures were taken, and both turned out to be negative. Antibiotic treatment was initiated with meropenem 3x2g IV and vancomycin 2x1g IV.

**Table 1:** Inflammation markers in serum along the course of treatment with dual approach colistin NR, no result

Date	CRP in serum, mg/L	Procalcitonin in serum, ng/L	WBC, 10^3^/mcL
4.10.23	10.3	0.17	NR
6.10.23	3.9	0.08	NR
7.10.23	32.3	5.58	43
8.10.23	245.0	4.61	21.26
9.10.23	182.0	2.46	19.57
10.10.23	197	1.39	19.21
11.10.23	180	0.72	9.33
12.10.23	164	0.50	10.92
13.10.23	160.0	0.32	11.07
14.10.23	142	0.44	11.14
16.10.23	91.9	0.26	7.64
17.10.23	45,9	0.17	7.58
19.10.23	13.7	0.11	4.54
20.10.23	7.9	0.07	6.06
21.10.23	5,7	0.06	8.25
22.10.23	9.5	0.08	9.66
23.10.23	8.7	0.07	11.05
24.10.23	NR	0.05	5.47
25.10.23	4.8	NR	5.03
26.10.23	4	0.04	7.97
27.10.23	2.8	0.04	6.83
28.10.23	NR	0.04	8.86
29.10.23	0,9	NR	10.89
30.10.23	<0.6	0.03	6.49
Normal range	<10	<0.49	4.00-10.00

**Figure 3:**
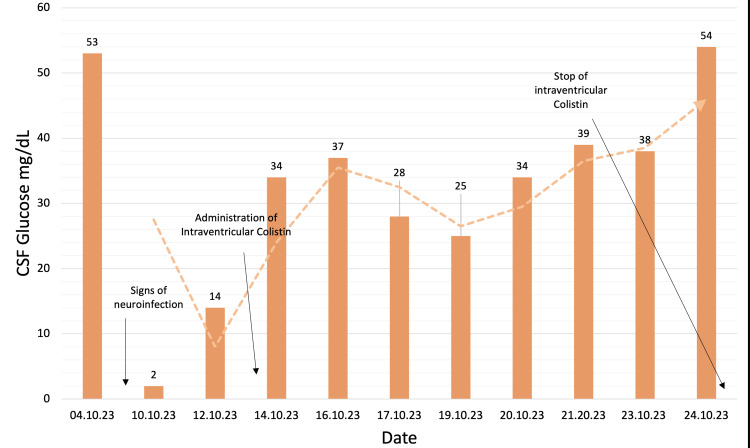
Glucose levels in CSF as a representation of successful treatment with a dual approach colistin administration CSF, cerebrospinal fluid

The next couple of days, the patient's condition deteriorated; he was tachycardic (124 BPM), and he had a mild fever of 37.3°C degrees. There was a further increase in CRP, WBC count, and procalcitonin (Figure 2, Table 1). Due to suspicion that the VP shunt is the source of the neuroinfection, it was decided to externalize it. During this procedure, 30 mL of murky CSF drainage from the spinal canal was evacuated, and cultures of the CSF were taken and sent for evaluation. The CSF culture returned, showing that the causative bacteria was AB. Daily administration of colistin 3 million units IV was added to the current antibiotic regimen consisting of meropenem and vancomycin IV. However, vancomycin was discontinued the day after. Total protein in CSF was significantly increased along with lactate, and glucose was almost non-existent (Figure 3, Table 2) with increased WBC and CRP (Figure 2, Table 1).

**Table 2:** Inflammation markers in CSF during dual approach therapy with colistin CSF, cerebrospinal fluid

Date	Protein, mg/dL	Glucose, mg/dL	Segmented cells, cells/µL	Lactate, mmol/L
4.10.23	46.6	53	19	3.7
10.10.23	301	2	30,006	13
12.10.23	173	14	1,653	8.6
14.10.23	147	34	169	8.1
16.10.23	136	37	97	7
17.10.23	483	28	Error	8.5
19.10.23	67.3	25	775	5.8
20.10.23	37.6	34	25	5.1
21.20.23	35.9	39	10	4.8
23.10.23	93.4	38	187	6.5
24.10.23	54.5	54	46	5.2
Normal range	20-40	40-75	<5	1.1-2.5

**Figure 4:**
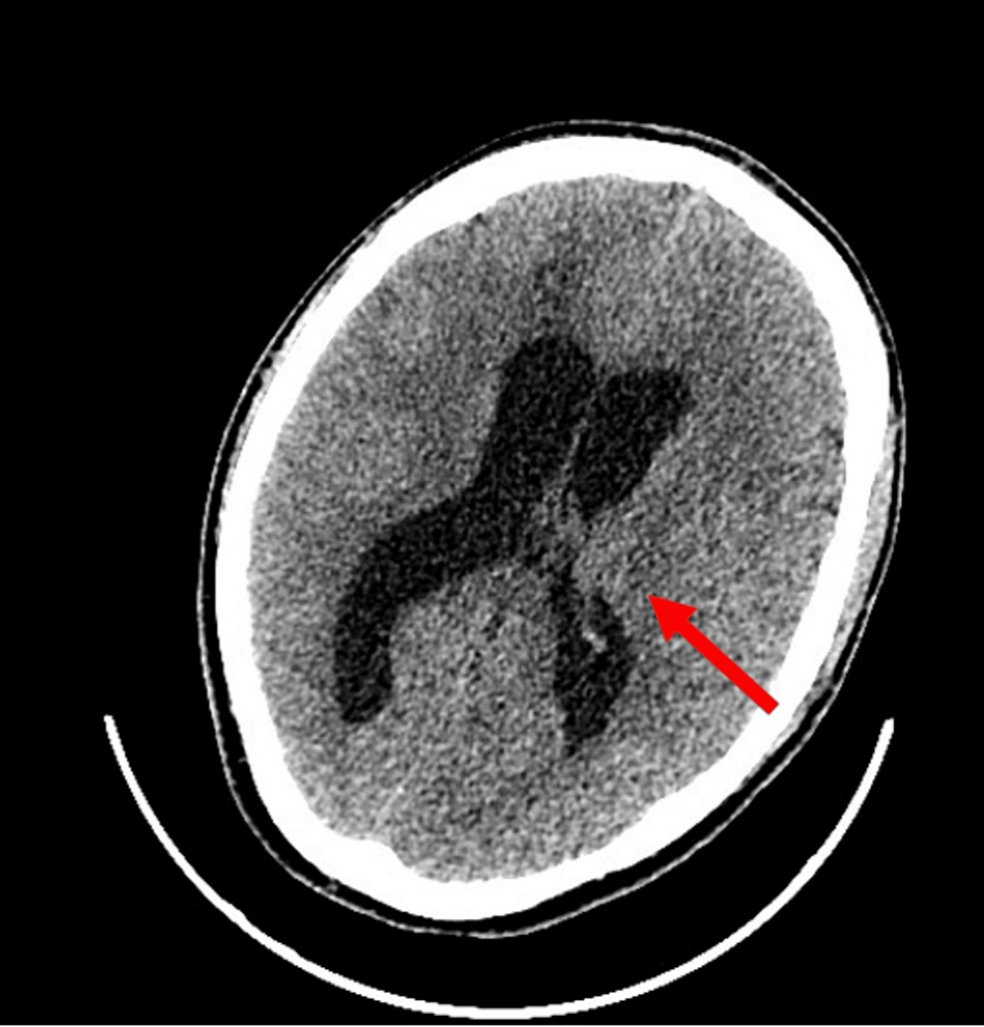
CT scan showing hydrocephalus on day 42 of the hospitalization

On day 42 of hospitalization, the patient's neurological status deteriorated. A CT scan revealed hydrocephalus, and immediate drainage was implemented (Figure 4). CSF analysis showed increased total protein, increased lactate, and decreased glucose (Figure 3, Table 2). The CSF culture's antibiogram revealed that this AB strain was susceptible only to colistin.

On the following day, day 43, for the first time, a dose of 1 million units (one-third ampule) dissolved in 2 mL of NaCL 0.9% was administered intraventricularly, while colistin IV and meropenem IV were resumed.

After two days of clinical improvement, it was suspected that the shunt had become clogged, and a new EVD was placed.

On day 46, three days after the initiation of the dual therapy with IV and IVT colistin, the patient experienced a myoclonic seizure. This seizure was successfully addressed with diazepam and levetiracetam. Despite a decrease in inflammatory parameters, the patient had a fever of up to 39.9°C. General urine and blood culture tests were negative, chest HRCT showed no signs of pneumonia, and abdomen CT showed no signs of peritonitis.

The patient received colistin IVT once daily for 14 days until day 57, when the decision was made to terminate the IVT colistin treatment due to a significant improvement in clinical signs and inflammatory markers. Throughout the treatment with colistin, the patient preserved normal kidney function with eGFR within the normal level. All inflammatory parameters showed improving dynamics until the end of the treatment with colistin and returned to normal levels.

**Figure 5:**
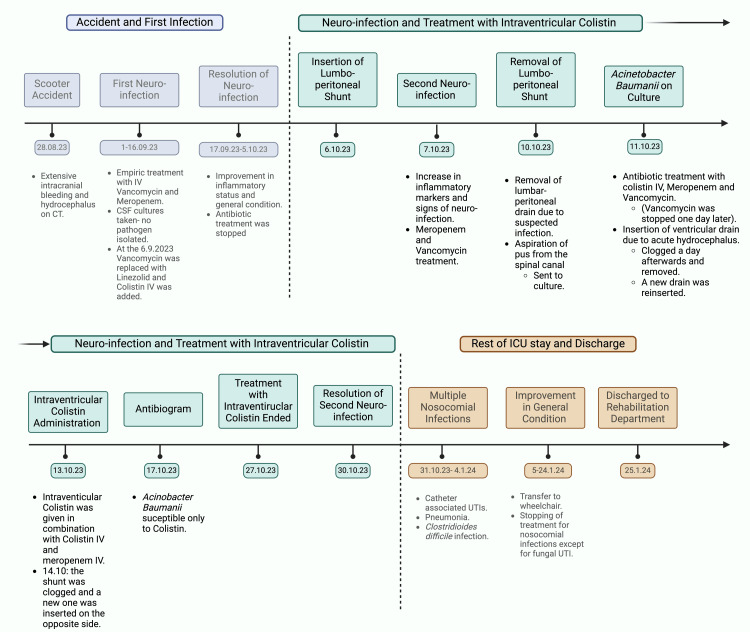
Timeline of stay and treatment of the patient with Acinetobacter baumanii ventriculitis in the ICU Image created with BioRender

After the resolution of the AB ventriculitis, the patient experienced different nosocomial infections during his stay in the ICU that were treated in accordance with the susceptibility of the cultured pathogens. The patient had a slow recovery, embedded with multiple nosocomial complications, but eventually was transferred to a wheelchair on day 127 of hospitalization with the support of his family (Figure 5). Eventually, the patient was transferred to a rehabilitation department after 147 days of ICU hospitalization.

## Discussion

Shunt-related infection is a severe complication of VP shunt application with devastating consequences for the patient. It is estimated that 7% of VP shunt operations result in an infection [9], with several modifiable risk factors [10,11]. In the described case, a young, healthy man needed rescue drainage and VP shunt due to traumatic hydrocephalus. Device-related infection occurred after exposure to multiple antibiotics. AB is a common causative pathogen in post-neurosurgical nosocomial infections [12]. Its emergence resulted in the development of various resistance patterns, usually subdivided into three categories: pan-drug-resistant (PDR), extensively drug-resistant (XDR), and MDR.

The nature of the causative pathogen, an XDR AB susceptible only to colistin, suggests that the infection was purely nosocomial and did not originate from gut flora. The patient was prophylactically treated with antibiotics before the insertion of the VP shunt, and all the prevention measures were taken. However, given the pathogen's resistance pattern, the prophylactic antibiotic regimen was inefficient. Therefore, we would like to raise awareness of XDR-pathogen nosocomial infections during surgical procedures that bypass the empiric prophylactic treatment, especially in ICU patients exposed to MDR pathogens.

Due to colistin's very poor penetration to the CSF in an IV route, with only 5% CSF-to-serum concentration in the IV form alone, the "International Consensus Guidelines for the Optimal Use of Polymyxins," colistin should be administered, when indicated, in both IV and IVT forms [13,14]. In this case, we faced an infection with XDR AB susceptible only to colistin (polymyxin E). De Bonis et al. show that a combined IVT and IV colistin is the most effective way to treat XDR AB neuroinfections [15]. In a literature review, a success rate of 89% was observed in the IVT or ITH administration of colistin in MDR AB during 83 episodes of ventriculitis in 81 patients [16]. In a study by Chen et al., 25 patients received IVT colistin, of which 20 patients were cured, and eight of these patients underwent previous shunt surgery due to hydrocephalus [17]. Colistin IVT has previously been shown to be superior to IV administration. The mean time of CSF sterilization has been found to be 21 days (range: 8-48 days) in a group of 18 patients [15]. In our case, colistin was given in an IV administration route for several days alone, with no significant clinical improvement. Therefore, we opted for dual therapy with IVT and IV, which resulted in complete recovery, showing that the combination approach was effective. The clinical results from our case are in accordance with the findings mentioned above, suggesting that the administration of IVT colistin was the reason for the improvement in the patient's condition.

The treatment with colistin is not trouble-free, with a high incidence of nephrotoxicity observed, mainly in early treatment [8]. Our patient did not present at any time of administration of colistin signs or laboratory results of renal dysfunction or failure. In addition to nephrotoxicity, neurotoxicity with the administration of colistin is commonly observed [18]. Our patient experienced a myoclonic seizure three days after the initiation of treatment with IV and IVT colistin. While the possibility that the cause of the seizure is the IVT administration of colistin is formidable, it cannot be confirmed. Our patient was in the middle of a neuroinfection and hydrocephalus after a severe head trauma, all of which are possible explanations for the seizure. Given that the diagnosis of colistin-induced neurotoxicity is mostly clinically based, we would like to emphasize the difficulty of determining the specific etiology of a seizure in a case of concomitant other neuropathologies.

## Conclusions

We believe that administering IVT and IV colistin in managing AB ventriculitis in susceptible instances has proven to be efficacious and relatively safe. We believe that this treatment plan can be applied to other future cases due to the positive clinical outcomes we observed. Given multiple possible underlying triggers, determining the etiology of myoclonic seizures can be challenging in this patient. However, colistin's IVT administration may have been a contributing factor.

Moreover, healthcare professionals must receive appropriate education regarding the risks associated with surgical-nosocomial XDR infections and the preventative measures that can be taken to avoid such infections. The emergence of AB infections in the ICU has become a matter of increasing significance due to the ability of AB to overcome frequently used prophylactic and empiric antibiotic regimens due to varying resistance mechanisms and patterns.
